# Recent progress in the research of cold-inducible RNA-binding protein

**DOI:** 10.4155/fsoa-2017-0077

**Published:** 2017-10-04

**Authors:** Peng Zhong, He Huang

**Affiliations:** 1 Department of Cardiology, Renming Hospital of Wuhan University, Wuhan 430060, PR China; 2 Cardiovascular Research Institute, Wuhan University, Wuhan 430060, PR China; 3 Hubei Key Laboratory of Cardiology, Wuhan 430060, PR China; 4 Department of Medicine, Johns Hopkins School of Medicine, Baltimore, MD 21287, USA

**Keywords:** cancer, cold-inducible RNA binding protein, inflammation, mRNA stability

## Abstract

Cold-inducible RNA-binding protein (CIRP) is a cold-shock protein which can be induced after exposure to a moderate cold-shock in different species ranging from amphibians to humans. Expression of CIRP can also be regulated by hypoxia, UV radiation, glucose deprivation, heat stress and H_2_O_2_, suggesting that CIRP is a general stress-response protein. In response to stress, CIRP can migrate from the nucleus to the cytoplasm and regulate mRNA stability through its binding site on the 3′-UTR of its targeted mRNAs. Through the regulation of its targets, CIRP has been implicated in multiple cellular process such as cell proliferation, cell survival, circadian modulation, telomere maintenance and tumor formation and progression. In addition, CIRP can also exert its functions by directly interacting with intracellular signaling proteins. Moreover, CIRP can be secreted out of cells. Extracellular CIRP functions as a damage-associated molecular pattern to promote inflammatory responses and plays an important role in both acute and chronic inflammatory diseases. Here, we summarize novel findings of CIRP investigation and hope to provide insights into the role of CIRP in cell biology and diseases.

## Lay abstract

Cold-inducible RNA-binding protein is a protein that can be be regulated by a variety of stress factors, such as cold temperature and UV radiation. Its response to stress is implicated in multiple cellular process such as cell proliferation, cell survival, circadian modulation, telomere maintenance and tumor formation and progression. It can also travel outside the cell where it can be involved in inflammatory diseases. This review summarizes our current knowledge of cold-inducible RNA-binding protein and its actions, what we have yet to understand and its potential for use in treatment of diseases ranging from cancer to diabetes.

Cold-inducible RNA-binding protein (CIRP), also called CIRBP or A18 hnRNP, encodes an 18-kD protein consisting of an N-terminal RNA recognition motif (RRM) and a C-terminal arginine-rich region (Figure 1), which exhibits structural similarity to a class of stress-induced RNA-binding proteins, that are predominantly found in the nucleus and involved in several cellular activities ranging from transcription and mRNA processing in the nucleus to cytoplasmic mRNA translation and turnover. CIRP cDNA was first cloned and characterized in mice [1], then in humans [2], and later in rats [3] and amphibians such as *Xenopus laevis* and bullfrogs [4]. Human CIRP cDNA also encodes an 18-kD protein whose amino acid sequence is 95.3% similar to that of the murine form and the *CIRP* gene is mapped to human chromosome 19, region p13.3 [2]. The rat CIRP is 100% identical to murine CIRP [3]. Bullfrog CIRP is 72.7% similar to mammalian CIRPs and 78.54% to *Xenopus laevis*’ CIRP [4]. The amino acid sequence of CIRP in different species is shown in Figure 1B. CIRP is ubiquitously expressed in a large variety of tissues and cells from both human and murine origins, including the brain, lungs, heart, kidneys, retinas, testis, liver, ovaries, nervous system, lymphocytes and endometrium [5]. CIRP is mainly localized in the nucleus, but can translocate to the cytoplasm under stress conditions. The nucleocytoplasmic distribution of CIRP is controlled by its C-terminal domain and is regulated by methylation [6,7]. Such a shift in the subcellular localization of CIRP might indicate a changing role of the protein over time. This review discusses the regulation, and physiological and pathological roles of CIRP as well the underlying mechanisms of processes in which CIRP is involved.

**Figure 1.**
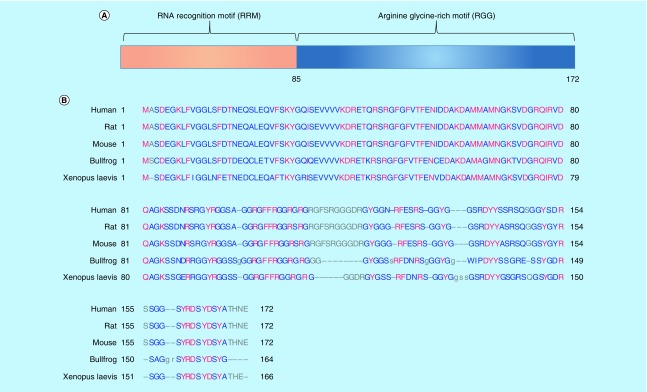
**The structure of CIRP.** **(A)** The schematic structure of CIRP is shown, which contains an N-terminal RRM) and C-terminal RGG; The RNA-binding domain is composed of about 85 amino acids. **(B)** Amino acid sequence alignment and comparison of amino acid sequences of CIRP from different species. The CIRP protein sequence of human (Q14011), rat (Q60825) and mouse (P60824), bullfrog (Q9PTX2), xenopus laevis (093235) were acquired from Universal Protein Resource (Uniprot) databases. CIRP: Cold-inducible RNA-binding protein; RGG: Arginine-rich region; RRM: RNA recognition motif.

## The regulation of CIRP in response to stimulation

### Factors regulating CIRP expression

**Figure 2.**
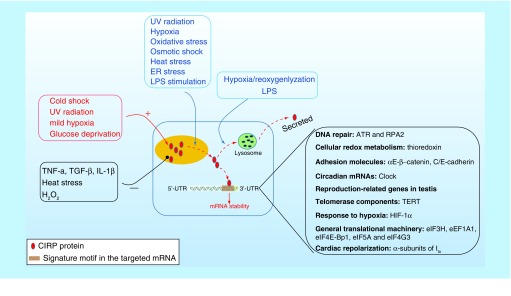
**The cellular response of intracellular CIRP and its role upon stress.** CIRP transcription and expression can be affected either upregulated or downregulated in response to various stress. CIRP is predominantly localized in the nucleus but can migrate to cytoplasm upon stress condition, and acts as an RNA chaperone regulating mRNA stability through its binding signature site in the 3′-UTR of its targets, which includes genes involved in DNA repair (*ATR*, *RPA2*), cellular redox metabolism (*thiroredoxin*), adhesion molecules (*αE/β-catenin*, *C/E-cadherin*), circadian mRNA (*clock*), reproduction-related genes in testis and TERT, response to hypoxia (*HIF-1α*), general translational machinery (*eIF3H*, *eEF1A1*, *eIF4E-Bp1*, *eIF5A*, and *eIF4G3*), and cardiac repolarization (α-subunits of I_to_). In addition, CIRP can also be secreted into extracellular space through lysosome pathway upon stimulation by LPS or hypoxia/reoxygenation. HIF-1α; Hypoxia-inducible factor 1α; TERT: Telomerase component.

CIRP upregulation is consistently observed in various organs upon mild hypothermia or cold stress in different species from amphibians to humans [8], which may be involved in adaptation in response to cold stress, suggesting that CIRP is a conserved cold stress protein among different species. In addition, CIRP is also upregulated upon UV radiation, mild hypoxia and glucose deprivation, but is decreased in response to heat stress and inflammatory cytokines including TNF-α and TGF-β [9], suggesting that CIRP is a more general stress responsive protein (Figure 2). Although, CIRP is upregulated upon mild hypoxia, chronic hypoxia has been reported to depress CIRP expression [10]. The influence of H_2_O_2_ on CIRP expression is controversial with some studies showing downregulation and others showing no change [3,11,6]. Activation of GSK3β can also increase the mRNA level of CIRP [12]. CIRP upregulation can also be regulated by IGF1 [13]. It should be noted that CIRP homologs in different species may have responded differently. For example, the transcription of the CIRP homolog in salmon can be upregulated by osmotic stress, but not by cold stress [14]. Various homologs of CIRP in Xenopus cells have been identified, including xCIRP, xCIRP-1 and xCIRP-2. Cold stress can upregulate xCIRP and xCIRP 2, but not xCIRP-1 [7,15,16]. Collectively, these results suggest that CIRP is a general stress protein and variation exists in its response between species.

### Mechanism of how CIRP is regulated

Three major CIRP transcripts with different transcriptional start sites, differing mainly in the size of their 5′-UTRs, have been identified with the levels of each of these transcripts being regulated by cold stress [17]. One transcript has the shortest 5′-UTR and is expressed at 37°C. The other two transcripts are expressed at 32°C, harbor larger 5′-UTRs and have shown internal ribosome entry segment-like activity, which allows mRNA to continue to be efficiently translated under stress when cap-dependent translation is diminished [17]. A further study identified an enhancer in the 5′ flanking region of the *CIRP* gene, which is termed mild cold responsive element that can be bound by transcriptional factor Specificicity protein 1 (Sp1) to activate gene transcription during mild hypothermia [18]. Upon mild hypothermia, Sp1 can be induced and accumulated in the nucleus to bind to the CIRP regulatory region containing mild cold responsive element. Overexpression of Sp1 can increase expression of CIRP, while downregulation of Sp1 has the opposite effect [18]. Sp1 is a general transcription factor which binds to and acts through GC-rich elements such as GC boxes, and is involved in the expression of many genes [19]. As Sp1 can also be induced by hypoxia [20], it is possible that hypoxia-induced CIRP transcription may also be mediated by Sp1. Whether or not Sp1 is expressed upon oxidative stress is controversial. Sp1 expression and activity have been reported to be suppressed or promoted in response to hydrogen peroxide treatment [21,22]. Further study found that splicing efficiency, which defined as the fraction of CIRP pre-mRNA processed into mature mRNA is involved in controlling CIRP expression in response to temperature fluctuations [23]. Gotic *et al.* demonstrated that although the steady-state levels of the mature CIRP mRNA increased significantly in response to mild cold exposure, the level of CIRP pre-mRNA did not change, and neither did the RNA polymerase II occupancy on the CIRP promoter or gene body, which argues against a transcriptional response [23]. Taken together, these results suggest that the regulation of CIRP expression in response to stress may be coordinated at multiple levels such as transcriptional activation, alternative splicing and splicing efficiency, either independently or collaboratively. More recently, a study using hypothermia-treated cells suggested a role of intracellular Ca^2+^ levels in regulating CIRP expression in response to cold stress [24], suggesting that intracellular calcium signaling may be involved in CIRP regulation.

### Factors regulating CIRP translocation

In addition to being regulated at transcriptional and protein levels, subcellular translocation of CIRP from the nucleus to cytoplasm is also observed under various stress conditions and shows stress-specific regulation (Figure 2). Specifically, upon cytoplasmic stress including oxidative stress, ER stress, osmotic shock and heat shock, CIRP can be methylated and migrates from the nucleus to stress granules (SGs), a kind of RNA granules [25], that are cytoplasmic foci in which untranslated mRNA can be sequestered [6], arguing for a general role of CIRP in SG physiology. However, cold shock markedly induces CIRP synthesis in various cell types but fails to induce the accumulation of CIRP in SGs [6]. UV irradiation, which also induces SGs, promotes CIRP nuclear export without further assembly into SGs [6]. These observations reveal the variability of CIRP expression and localization in response to different types of stress, the potentially different roles CIRP might play in response to various stressors, and the diverse mechanisms by which CIRP is regulated.

Post-translational modification of CIRP has been shown to regulate its cellular localization as well as its RNA binding activity. Methylation of CIRP is required for its cytoplasmic translocation, as cytoplasmic translocation of CIRP can be inhibited by a methyltransferase inhibitor or enhanced by overexpression of methyltransferase [6,7]. Phosphorylation of CIRP by GSK3β and CK2 could also affect its cellular localization, as pretreatment of cells with either CK2 inhibitors or GSK3β inhibitors prior to UV exposure is sufficient to significantly reduce CIRP translocation to the cytosol, with the effects of CK2 inhibitor being more pronounced [26]. These results suggest that both methylation and phosphorylation of CIRP may be required for CIRP cytoplasmic translocation upon stress.

In addition to regulating its cellular distribution, phosphorylation of CIRP has also been involved in regulating its activity. Although, CK2 seems to be the predominant kinase involved in CIRP translocation, it does not affect its RNA-binding activity [27], while phosphorylation of CIRP by GSK3β increases its transcript binding efficiency twofold [27]. It seems that both CK2 sites and GS3Kβ sites on CIRP may have overlapping role in regulating CIRP cytoplasmic translocation, but only GSK3β sites are involved in the RNA-binding activity of CIRP, in response to UV radiation. Because hypoxia can also induce GSK3β and CK2 [28,29], it seems likely that under hypoxic conditions, CIRP may be phosphorylated by GSK3β and CK2 and then translocated to the cytosol.

## The RNA-dependent function of CIRP: a cell autonomous function

### RNA exerts its function through its RNA-binding activity

CIRP exerts its function by preferentially targeting translation of specific mRNA transcripts harboring its RNA signature motif in response to cellular stress (Figure 2). In the cytosol, CIRP binds to the 3′-untranslated region (3′-UTR) of RNA transcripts on ribosomal fractions and increases the mRNA stability, consequently enhancing translation [26,27,30,31]. Later, the CIRP-binding motif in the 3′-UTR of CIRP mRNA targets is identified, which consists of a 51-nucleotide RNA motif [26]. The 3′-UTR binding sites of CIRP are enriched within 100 nucleotides upstream of the polyadenylation sites [32]. Further study validated that UU and UUU are the possible core recognition sequences of CIRP [31]. As the binding of CIRP to mRNA is mediated in part through the RRM, the x-ray crystal structure for the RRM of CIRP was reported recently and four residues were identified as being likely involved in protein–nucleic acid interactions, which may help to serve as a foundation for biophysical studies of this RNA-binding protein and structure-based drug-design efforts for targeting CIRP in pathological conditions, where CIRP levels are elevated and contribute to disease progression [33].

Currently, CIRP has been demonstrated to positively and post-transcriptionally regulate genes involved in DNA repair [34,26,30], cellular redox metabolism [27], adhesion molecules [35], circadian mRNAs [36], reproduction-related genes in testis [31], telomerase components [37], HIF-1α [38] and a number of transcripts associated with the general translational machinery [38]. In addition to the positive post-transcriptional regulation, a negative role of CIRP in translation has also been reported. Upon stress, CIRP migrates from the nucleus to cytoplasmic SGs and acts as a translational repressor in Cos, Hela, NIH3T3 and 293T cells [6]. It was also demonstrated that CIRP could post-transcriptionally and negatively modulate expression of the α-subunits of I_to_ channels in cardiomyocytes, affecting cardiac repolarization [39].

CIRP may regulate mRNA stability by inhibiting deadenylation of its target mRNAs, maintaining elongation of poly(A) tail length, and subsequently translational activity [40]. CIRP from both mammalian and Xenopus cells has been demonstrated to be associated with human antigen R (HuR), which can positively regulate the stability and translation of mRNAs by interacting with AU-rich element, that are located in the 3′-UTR of many mRNAs [41,40,42]. In addition, CIRP can also enhance HuR expression and its activity [40,42]. These results suggest an important role of HuR in mediating the translational regulation of CIRP. Furthermore, CIRP may regulate translation of its targets at the initiation phase. CIRP can associate with low molecular weight polysomes and interact with eIF4G [27], suggesting that CIRP can bridge its targeted transcripts to the general translational machinery through interactions with eIF4G [27]. Besides, its role in regulation mRNA translation and stability control in the cytoplasm, nuclear CIRP has also been reported to be implicated in post-transcriptional regulation of its targets by controlling alternative polyadenylation, which is an important mechanism in the post-transcriptional regulation that generates mRNA with alternative 3′ ends [32]. Collectively, these results suggest a multiple role of CIRP in control post-transcriptional regulation either by controlling alternative polyadenylation of its target or regulating mRNA stability or accelerating translational initiation.

### The potential targets of CIRP revealed by multiple high-thoughput screening methods

*In vitro* RNA–protein interactions in a nunc-immuno tubes assay show that a large proportion of isolated mRNAs from cells exposed to UV radiation, which can bind with recombinant CIRP, are stress activated [30]. Further studies exploring the molecular mechanism of CIRP by using genome-wide expression microarrays in CIRP knockdown cells and pathway analyses of differentially expressed genes in CIRP-deficient cells and control cells showed five enriched pathways, namely, focal adhesion, MAPK, Wnt, apoptosis and cancer-related signaling pathways, which may be the central pathway networks regulated by CIRP [43]. An RNA-binding protein immunoprecipitation assay in mouse testis revealed that most of the CIRP mRNA targets are associated with translation regulator activity, antioxidant activity, envelopment and reproduction, and that mRNAs bound to CIRP increase their stability [31]. Through the UniGene database, it was found that the CIRP RNA binding signature motif could be located in the 3′-UTRs of transcripts associated with proliferation, survival and invasion [26,38]. Gene ontology analyses of CIRP-interacting and -regulated transcripts unveiled an enrichment in functions related to cell-cycle progression and adhesion [36]. By immunoprecipitation and RT-PCR, a panel of adhesion molecules including αE- and β-catenin, C-and E- cadherin and paraxial proto-cadherin has been identified as the targets of CIRP in Xenopus [35]. These results suggest broad targets and multiple roles of CIRP in cell biology.

### Role of CIRP in regulating spermatogenesis

In the mouse testis, CIRP is constitutively expressed in the germ cells and the level varies depending on the stage of differentiation [44]. CIRP is expressed in primary spermatocytes, secondary spermatocytes and round spermatids, but is not expressed in spermatogonia, elongating spermatids, Sertoli cells or Leydig cells [44]. These results suggest that CIRP may have a role in spermatogenesis. However, CIRP^−/−^ mice are viable, reproductive and fertile and display no obvious phenotype under normal laboratory conditions [7,45]. Interestingly, CIRP-deficient mice showed a reduction in the number of undifferentiated spermatogonia, and the rate of recovery of spermatogenesis was affected when they were exposed to a cytotoxic agent, compared with wild-type mice [45]. These results suggest a nonessential but a growth-stimulatory role of CIRP in spermatogenesis. A mechanistic study found that CIRP can accelerate cell-cycle progression by binding to Dyrk1b and preventing it from phosphorylating both p27 and cyclin D1, leading to p27 destabilization and cyclinD1 stabilization at the protein level, which are required for effective progression of the cell cycle to S phase [45]. These results suggest a role of CIRP not limited to regulating mRNA stability.

### Role of CIRP in regulating circadian rhythm

CIRP may have a role in regulating circadian rhythm in amphibians and mammals, as circadian expression of CIRP was observed in the brains of both Xenopus laevis [4] and mice [46]. Expression of brain CIRP is also affected by sleep state, with increased expression in the rested state, and downregulation in the sleep-deprived state [47]. Further mechanistic studies found that the rhythmic expression of CIRP is controlled by body temperature cycles and CIRP is required for high-amplitude circadian gene expression *in vitro* [36]. CIRP can directly interact with many of the circadian mRNAs, including clock, and the loss of CIRP reduces the amount of cytoplasmic Clock mRNA and protein throughout the day, and mimics the effects observed in Clock depletion, with a decrease in mRNA of many core circadian components including Clock, Ncor1, Per2, Per3 and Dbp [36]. These results suggest that CIRP can enhance the amplitude of circadian oscillators by increasing the cytoplasmic accumulation of mRNAs encoding clock and perhaps, other regulators relevant for the circadian timing system, such as RORα, NCOR1, SIRT1 and PER3 [36].

Subsequent studies further suggest CIRP as an important mediator in regulating circadian rhythm by controlling circadian gene expression in response to environmental factors. CIRP may be responsible for ketogenic diet or fasting-induced robustness of peripheral oscillators in the liver *in vivo*, as liver CIRP expression was increased and associated with enhanced magnitude and amplitude of circadian gene expression [48]. In addition, CIRP has also been demonstrated to mediate the downregulation of clock genes induced by inflammatory cytokines such as TNF-α and TGF-β [9]. TNF-α and TGF-β could impair the expression of CIRP, while IL-1β, IL-6, IFN-α and IFN-γ do not exert such effects. Depletion of CIRP is found to increase the susceptibility of cells to TNF-α-mediated inhibition of high amplitude expression of clock gene expression [9]. Upregulation of CIRP is also found to be associated with circadian reprogramming and cancer inhibition induced by meal timing, compared with that in mice fed *ad libitum*, suggesting that CIRP may be a putative candidate to integrate circadian restrains into tumor growth [49]. Taken together, these results indicate that CIRP may act as a nodal signal connecting environmental signal to circadian regulation.

### Role of CIRP in regulating proliferation & differentiation

CIRP upregulation has been reported to be involved in cold-induced growth suppression of mouse fibroblasts NIH/3T3 cell line by prolongation of G1 phase [1]. CIRP expression intensity in glandular cells of human endometrium, is inversely proportional to its proliferative activity during the menstrual cycles [50]. Overexpression of CIRP in the mammary epithelium was reported to suppress proliferation of mammary glands during the lactational switch (the transition from pregnancy to lactation) in mammary glands [51]. Collectively, these results suggest a negative role of CIRP in cell proliferation.

By contrast, CIRP can accelerate cell-cycle progression from G0 to G1 as well as from G1 to S phase in cultured mouse embryonic fibroblasts and spermatogonial cell line [45]. Overexpression of CIRP also enhance cell proliferation in the mammalian kidney cells [43]. Decreased CIRP was also reported to mediate proliferation inhibition induced by severe hypoxia in neural stem cells, which can be restored by forced expression of CIRP [52]. These results suggested a positive regulatory role of CIRP in cell growth. However, overexpression of CIRP has no effects on cell growth in recombinant Chinese hamster ovary cells [53,54] and human colorectal carcinoma RKO cells [30]. Taken together, these results suggest that the role of CIRP on cell growth may be cell-type dependent, and further studies are needed to elucidate the vastly differing outcomes of CIRP underlying cell proliferation.

In addition to being involved in cell growth, CIRP also plays a role in cell differentiation in Xenopus. Three *CIRP* isoforms has been identified in Xenopus cells including *xCIRP*, *xCIRP1* and *xCIRP2*. *xCIRP* and *xCIRP1* mRNA is to 98% identical resulting in proteins of 99.4% identity and xCIRP protein is to 94% identical to xCIRP2, implicating that these isoforms derived from different alleles of pseudotetraploid in Xenopus [55]. xCIRP is highly expressed at a particular stage of pronephros formation at an early developmental stage of Xenopus embryo, with its function unrevealed [56]. xCIRP1 plays keys role in the differentiation and morphogenesis during early development of Xenopus laevis, as microinjection of antisence RNA targeting xCIRP1 into Xenopus embryos produces tailbuds with deformation of brain and internal organs [7,15]. xCIRP2 is a major cytoplasmic protein in Xenopus oocytes, which can bind to mRNA and associate with ribosomes, suggesting a possible role involved in translational regulation through the modulation of ribosomal function [16]. These results suggest that CIRP plays key roles in differentiation and morphogenesis during early development in Xenopus.

### Role of CIRP in regulating cell survival & apoptosis

Accumulating studies showed a protective role of CIRP in regulating cell survival and cell apoptosis against harmful stress in various kinds of cells including germ cells, neural cells, cardiac cells and stem cells. Induction and cytoplasmic translocation of CIRP plays a protective role against genotoxic stress upon UV radiation, by stabilizing specific transcripts involved in cell survival [30]. CIRP is also demonstrated to be a regulator of p53-dependent apoptosis pathway induced by DNA damage, in which overexpression of CIRP can downregulate p53, while knockdown of CIRP exacerbates p53 expression in response to DNA damage [57].

Downregulation of CIRP increases germ cell apoptosis, and is associated with heat stress-induced germ cell apoptosis [58,59], contributes to chronic hypoxia-induced neuron apoptosis [10], and promotes ischemia-induced cell apoptosis in cardiac cells *in vitro* [60], while overexpression of CIRP protects testes damage and cell apoptosis induced by cryptorchidism or ischemia/reperfusion [61,62], reduces ischemia-induced cardiac cell apoptosis *in vitro* [10], and bypasses replicative senescence in primary cells [63]. In addition, hypothermia-induced CIRP protects cell apoptosis in TNF-α-treated BALB/3T3 cells [64], contributes to the preservation of the stemness of neural stem cells [65], prevents cell apoptosis upon EGF deprivation [65], and inhibits cell apoptosis in primary rat cortical neurons in response to H_2_O_2_ treatment [66] or long-term cell culture *in vitro* [67]. CIRP induction upon therapeutic hypothermia was also reported to improve neurological outcomes, and inhibited mitochondrial apoptosis in the cardiac arrest rat model [68] and in traumatic brain *in vivo* [69]. Taken together, these studies demonstrated a critical role of CIRP in regulating cell survival and protecting against cell apoptosis.

### Role of CIRP in telomere maintenance

Mammalian telomeres are essential terminal structures for maintaining genome integrity and stability. Telomeres are elongated and maintained by the telomerase. The mammalian telomerase core complex consists of the catalytic subunit TERT and the RNA component TERC, where the TERC RNA serves as a template for TERT-mediated telomere elongation. Recently, CIRP was identified as a new telomerase regulator and played a crucial role in telomere maintenance [37]. CIRP is identified as a telomerase-interacting factor through immunoprecipitations coupled with mass spectrometry and CIRP is necessary to maintain telomerase activity at both 32 and 37°C. Inhibition of CIRP by CRI surface plasmon resonance-Cas9 or siRNA knockdown led to reduced telomerase activity and shortened telomere length [37]. CIRP associates with the active telomerase complex through direct binding of TERC and regulates localization of the telomerase in Cajal body, where telomerase assembly and TERC maturation take place. Furthermore, CIRP regulates the level of *TERT* mRNAs, by which TERT is upregulated at lower temperature, to compensate for reduced telomerase activities [37]. *TERT* expression and telomerase activities are low or undetectable in most human somatic cells. However, telomerase activation is evident in ≥85% of cancers [70], suggesting that upregulation of *TERT* expression and telomerase activation is a key step in cancer development. Therefore, studies on the control of telomerase expression and activity are crucial to our understanding of the pathways that lead to cellular transformation and carcinogenesis. The identification of CIRP as a telomerase and telomere regulator provides new avenues to explore previously unknown mechanisms of telomerase control and telomere maintenance.

### Role of CIRP in tumorigenesis & malignant transformation

Changes of CIRP expression are observed in various cancer tissues. CIRP is upregulated in a subgroup of patients with solid cancers including colon cancer, prostate cancers, central nervous system-related tumors and liver-pancreas carcinomas, in which, CIRP is expressed mainly in the cytoplasm [63], suggesting that CIRP upregulation and translocation may have biological significance in human tumors. The cytoplasmic translocation of CIRP is also observed in human squamous cell carcinoma [71]. The cytoplasmic localization of CIRP in tumors is consistent with the fact that most solid tumors develop hypoxic regions, and CIRP translocates to the cytosol in response to hypoxia [26,72]. Later, the general characteristically upregulation of CIRP in tumors is further conformed by Chang et al., who found that CIRP is overexpressed in 40–60% of malignant tissues such as human melanoma, prostate, breast and colon cancers, compared with normal adjacent tissue [38]. Interestingly, in two mouse xenograft models including melanoma and breast cancer, downregulation of CIRP decreased tumor growth, proliferation, invasion and migration, which was associated with decreased protein translation of selected transcripts that are implicated in angiogenesis and translation machinery [38]. These results suggest an important and universal role of CIRP in the development of tumor progression. In addition to being present intracellularly, CIRP is also present in the stroma of several cancer tissues [38], suggesting that under certain conditions CIRP may be secreted and could potentially contribute to maintain cancer progression and/or propagation, which remains to be determined. On the contrary, expression of CIRP is reported to be markedly reduced in most of human endometrial carcinomas [50], which may need further confirmation because of the generally upregulation character of CIRP in cancer.

Clinical correlation studies indicate that CIRP may have the potential to be a prognostic biomarker for cancer progression. High level of CIRP in human oral squamous cell carcinoma is associated with a short survival rate [73]. CIRP expression level is also positively associated the invasive ability of pituitary adenoma in human [74]. An integrated analysis of serum autoantibody expression profiling in patients with breast cancer, found that serum autoantibody targeting CIRP was significantly increased in patients with breast carcinoma and its change can be used as a prognostic biomarker for cancer progression [75]. Although, the prognostic role for CIRP is suggested, more large sample size and different cancer types are needed to confirm this.

*In vitro* studies using various cancer cell lines also suggest that CIRP promotes cell proliferation and invasion in cancer cells. Elevated CIRP is observed in melanoma and nonmelanoma skin cancer cell lines, and confers resistance of keratinocytes to UVB-induced growth arrest and death [76]. CIRP upregulation promotes cell proliferation in pituitary corticotroph adenoma via ERK signaling pathway [77]. Overexpression of CIRP has been reported to promote epithelial–mesenchymal transition, a critical component of carcinoma metastasis and invasion [78]. On the other side, CIRP downregulation has been reported to be associated with effective chemotheraphy in prostate cancer cells [79,80], and associated with proliferation inhibition in Hela and TERA2 cells [63]. Collectively, these results strongly suggest an oncogenic role for CIRP in cancer progression.

As the upregulation of CIRP has been observed in a considerable number of cancers with capability of promoting cell proliferation, and can be induced in hypoxic conditions that are present in tumor environment, CIRP has been regarded as a new generation of proto-oncogene [81].

## RNA-independent function of CIRP: a noncell autonomous function

### Role of CIRP in regulating inflammation & inflammation-related diseases

CIRP plays a critical role in mediating inflammatory process. Knockdown of CIRP decreased the inflammatory cytokine IL-1β expression by inhibiting NF-κB signaling pathway in response to UV exposure or LPS stimulation [82], suggesting that the function of CIRP may be involved in inflammatory process. Later, the protein level of CIRP is found to be increased in the serum and organs in systemic inflammation-related diseases such as sepsis and hemorrhagic shock in both human and animal models, and mice lacking *CIRP* or treated with a neutralizing antibody to CIRP, showed markedly improved survival after hemorrhagic shock and sepsis [83]. Mechanistic study found that, in response to hypoxia/reoxygenation stress or LPS stimulation, CIRP can be induced and translocate from nucleus to cytosol and then be released to extracellular space by lysosomal secretion in macrophages cells [83]. Recombinant mouse or human CIRP (rmCIRP or rhCIRP) can induce the release of proinflammatory cytokines including TNF-α and HMGB1 in a dose- and time-dependent manner from cultured macrophages cells, and *in vivo* administration of rmCIRP to healthy rats increased serum TNF-α, IL-6 and HMGB1 levels and induced liver injury, as assessed by increased levels of the organ injury markers [83]. Furthermore, CIRP-induced inflammatory response is dependent on TLR4, and *in vitro* surface plasmon resonance analysis validated the physical interaction between CIRP and TLR4-MD2 complex [83]. Taken together, these results demonstrated that extracellular CIRP is an endogenous proinflammatory mediator and damage-associated molecular pattern (DAMP) that triggers inflammatory responses during hemorrhagic shock and sepsis [83].

In addition to triggering inflammatory cytokines expression in macrophage, extracellular CIRP is also reported to induce mitochondrial DNA (mtDNA) fragmentation and subsequent necroptosis in macrophage [84]. CIRP was found to be an important factor in the damaged tissue by trauma and could induce mtDNA fragmentation in various source of macrophages including alveolar macrophages, peritoneal macrophages and bone marrow-derived macrophages, through TLR4-MyD88-NADPH oxidase-ROS pathway, which in turn served as a major mediator for the induction of endonuclease G, an apoptotic DNase located in the mitochondrial intermembrane space, that directs mtDNA fragmentation [84]. Furthermore, the fragmented DNA can be released by necroptosis and worked as DAMP to promote inflammation in normal macrophages [84]. Collectively, these results suggested a versatile role of CIRP in regulating inflammation through macrophage.

**Figure 3.**
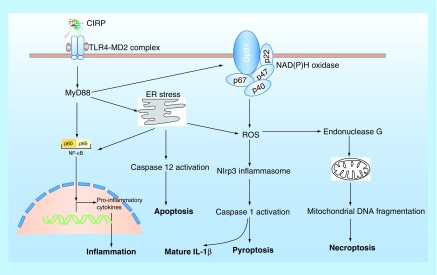
**The extracellular role of CIRP in cells.** Extracellular CIRP can trigger multiple effects in cells via TLR4-MyD88 signaling pathway. ER stress: endoplasmic reticulum stress; NADPH oxidase is a multimolecular enzyme, composed of a membrane-associated 22-kDa α-subunit (p22phox) and a 91-kDa β-subunit (gp91phox, with cytosolic components composed of p47phox, p67phox and p40phox. Endonuclease G is a mitochondrion-specific nuclease that located in mitochondria.

The release of CIRP can also induce vascular endothelial cell (EC) injury by activating Nlrp3 inflammasome pathway, leading to EC pyroptosis [85]. Yang et al. reported that in primary mouse lung vascular ECs, rmCIRP can increase cell-surface adhesion molecules expression and activate NAD (P) H oxidase and induce EC pyroptosis by activating Nlrp3 inflammasome [85]. Furthermore, intravenous injection of rmCIRP in C57BL/6 mice causes lung injury, evidenced by vascular leakage, edema, increased leukocyte infiltration and cytokine production in the lung tissue [85]. These results suggest that the released CIRP in shock can directly activate ECs and induce EC pyroptosis to cause lung injury. Induction of endoplasmic reticulum stress is also reported to be involved in sepsis/CIRP release-induced acute lung injury, which can be blocked in TLR4 KO or CIRP KO mice [86], suggesting that CIRP can regulate ER stress through TLR4 signaling pathway. In addition, the release of CIRP during sepsis is capable of initiating adaptive immune response by directly activating CD4^+^ and CD8^+^ T cells in TLR4-depentent manner in the spleen [87]. Later, the plasma level of CIRP of patients with sepsis is found to be significantly higher in the nonsurvivors than in the survivors, and independently predicts sepsis mortality [88], suggesting that CIRP may be a potent predictor of sepsis prognosis in human. Taken together, these results suggest that CIRP is an important mediator in organ dysfunction during sepsis by amplifying inflammation in macrophage and damaging vascular EC in all organs. Interestingly, a very recent study reported that a synthetic oligopeptide, called C23, which derived from the human CIRP region (Ser110-Glu125) with the highest affinity to rhMD2 [83], showed great potential against CIRP-induced phagocyte secretion of TNF-α, decreased activation of leukocytes and EC, and attenuated lung injury caused by hemorrhagic shock [89]. These results suggested a therapeutic potential of CIRP-targeting peptide in treating hemorrhagic shock. The extracellular role of CIRP is summarized in Figure 3.

In addition to mediating acute systemic inflammatory condition, CIRP is also involved other kinds of inflammation-related diseases. CIRP is reported to be an important mediator in alcohol-induced brain inflammation, and in cerebral ischemia-induced neuroinflammation, both of which are inhibited in CIRP knockout mice [90,91]. In cultured mouse microglia BV2 cells treated with either alcohol or hypoxia *in vitro*, CIRP can be induced, translocate and releases into culture medium, coupled with increased inflammatory cytokines expression and addition of recombinant murine CIRP directly induces TNF-α release from BV2 cells, which can be inhibited by neutralizing antisera to CIRP [90,91]. In addition, CIRP is found to be increased in hepatic ischemia-reperfusion mouse model and acts as a potent inflammatory mediator in liver, and blocking CIRP with antisera protects against I/R-induced liver injury [92]. Similarly, deficiency or blockage of CIRP also resulted in less renal injury in renal ischemia-reperfusion mice model [93]. Increased CIRP expression is also observed in the serum and aneurysmal tissues of human abdominal aortic aneurysms (AAA) and elastase-induced AAA rat, which is associated with increased expression of matrix metalloproteinase (MMP)-2, MMP-9, TNF-α and macrophage accumulation [94]. Interestingly, chronic treatment with CIRP neutralizing antibody significantly suppressed the dilation of experimental AAA and inhibited macrophage accumulation and MMP-2/9 expression [94]. In RAW264.7 macrophage cell line, recombinant mouse CIRP (rmCIRP) can directly increase MMP-9 expression and promote cell migration [94]. These results demonstrated a central role of CIRP in the pathogenesis of AAA.

Recently, CIRP is found to be highly expressed in the bronchi in patients with chronic obstructive pulmonary diseases and *in vitro* study found that CIRP contributed to cold stimulation-induced expression of inflammatory cytokines and mucin in human airway epithelial cells by activating ERK and NF-κB signaling pathway [95,96] suggesting that CIRP plays an important role in chronic airway inflammation diseases and mucus overproduction. In addition, CIRP concentration is also increased in the synovial fluid in patients with knee osteoarthritis and is associated with severity in osteoarthritis [97]. Collectively, these results suggest that upon diseased condition, CIRP may be released in the extracellular space, which may exacerbate tissue injury through inflammatory response.

In contrast, in mouse embryonic fibroblasts, CIRP-depletion augmented cytokine response to TNF treatment, in which 73 (77%) of 94 cytokine/cytokine receptor genes showed an increased expression [9]. In addition, CIRP deficiency improved cutaneous wound healing in mice by accelerating the inflammatory state and the resolution of inflammation, in which TNF-α mRNA level and neutrophil infiltration in wounded tissues were significantly increased in CIRP^−/−^ mice on day 3, but markedly decreased on day 7, which was followed by earlier phase of tissue formation and improved histological integrity, compared with WT littermates [98]. These results suggest a complex role of CIRP in mediating inflammatory process and may be in a cell- or tissue-specific manner.

### CIRP as a possible link connecting inflammation to tumorigenesis

Recently, studies suggest that CIRP plays an important role in linking inflammation to tumogenesis. In murine models of hepatocarcinogenesis induced by chronic treatment with thioacetamide or diethylnitrosamine, CIRP expression was increased in the liver and mainly expressed in liver macrophages called Kupffer cells and isolated Kupffer cells showed increased inflammatory cytokines such as IL-1β and IL-6 [99]. Interestingly, *CIRP* deficiency significantly decreased these inflammatory factors in isolated Kupffer cells and decreased tumor size and numbers in liver [99], suggesting that the proinflammatory role of CIRP in Kupffer cells contributed to thioacetamide/diethylnitrosamine-induced hepatocarcinogenesis. In addition, CIRP also plays an important role in colitis-associated cancer [100]. In human colonic muscosa of patients with ulcerative colitis, expression of CIRP correlated significantly with the expression of inflammatory cytokines (TNFa, IL23/IL17), antiapoptotic proteins (Bcl2, Bcl-xl) and stem cell markers (Sox2, Bmi1 and Lgr4) and the expression of CIRP was enhanced in the colonic mucosae of refractory ulcerative colitis. Interestingly, *CIRP* ^−/−^ mice exhibited decreased susceptibility to colonic inflammation induced by dextran sodium sulfate and reduced tumorigenesis in murine colitis-associated cancer model [100]. In addition, transplantation of *CIRP* ^−/−^ bone marrow into WT mice reduced tumorigenesis [100]. These results suggest that CIRP expression is upregulated by chronic inflammation in human and mice, enhances the inflammatory response and tumorigenesis by increasing Bcl2 and Bclx expression and TNF-α and IL23/IL17 production in inflammatory cells. Taken together, CIRP may be a critical mediator in linking inflammation to carcinogenesis.

## Conclusion & future perspective

Since first being identified as cold-response protein two decades ago, efforts have been put into elucidating the physiological and pathological biological role of CIRP. Currently, CIRP is widely distributed across almost every cell in mammalian and is a stress responsive protein that is likely contributing to various stress responses differently through a combination of changes in protein levels and nucleocytoplasmic shuttling. Multiple functions of CIRP have been identified via acting either intracellularly or extracellularly. Intracellular CIRP acts as an RNA chaperone, regulating mRNA stability through its binding sits on its targets, or transmit signals through interacting with other signaling proteins, thus regulating cell proliferation, cell survival, apoptosis and circadian rhythm, telomere maintenance and carcinoma progression. In addition, CIRP can be secreted out of cells and extracellular CIRP functions as DAMP that promotes inflammatory response, thus CIRP has been involved in various acute and/or chronic inflammatory diseases. Considering the widespread role in multiple kinds of cancer, CIRP has been regarded as a new oncogene, and has a great potential to be a therapeutic target in cancer treatment. Extracellular CIRP can also be detected in the matrix of several tumors, which raises a question on the role of extracellular CIRP in tumor microenviroment, so further study is needed to define this. The proinflammatory role and the broad involvement, and the apparent protective effects of CIRP blockage with CIRP neutralizing antibody in sepsis and other inflammatory diseases strongly demonstrate a critical role of CIRP in inflammation-related diseases and provide basis for the therapeutic potential of CIRP neutralizing antibody or CIRP-targeting peptide in treating inflammatory diseases. Although we have achieved great success in understanding the role of CIRP in cell biology and diseased conditions, there are still many questions unresolved, such as what is the role of CIRP in chronic inflammatory diseases such as obesity, diabetes and other kinds of diseases. Therefore, elucidating the role of CIRP in these diseases will have a significant impact on our understanding of (patho) physiology of diseases and may provide the rationale for the design of novel therapeutics.

**Executive summary**

- Cold-inducible RNA-binding protein (CIRP) is a stress-response protein, which is predominantly located in the nucleus and can be subjected to transcriptional regulation, cytoplasmic translocation, post-translational modification or secreted extracellularly upon stress condition.
- CIRP exerts its function either through its RNA-binding activity by binding its target genes intracellularly or acts as a secreted damage-associated molecular pattern triggering inflammatory response extracellularly.
- The role of CIRP has been implicated in multiple cellular processes such as cell proliferation, cell survival, circadian rhythm, telomere maintenance, tumorigenesis and malignant transformation and the inflammatory processes.

## References

[1] Nishiyama H, Itoh K, Kaneko Y, Kishishita M, Yoshida O, Fujita J (1997). A glycine-rich RNA-binding protein mediating cold-inducible suppression of mammalian cell growth. *J. Cell Biol.*.

[2] Nishiyama H, Higashitsuji H, Yokoi H (1997). Cloning and characterization of human CIRP (cold-inducible RNA-binding protein) cDNA and chromosomal assignment of the gene. *Gene*.

[3] Xue JH, Nonoguchi K, Fukumoto M (1999). Effects of ischemia and H2O2 on the cold stress protein CIRP expression in rat neuronal cells. *Free Radic. Biol. Med.*.

[4] Saito T, Sugimoto K, Adachi Y, Wu Q, Mori KJ (2000). Cloning and characterization of amphibian cold inducible RNA-binding protein. *Comp. Biochem. Physiol. B, Biochem. Mol. Biol.*.

[5] Larrayoz IM, Rey-Funes M, Contartese DS (2016). Cold shock proteins are expressed in the retina following exposure to low temperatures. *PLoS ONE*.

[6] De Leeuw F, Zhang T, Wauquier C, Huez G, Kruys V, Gueydan C (2007). The cold-inducible RNA-binding protein migrates from the nucleus to cytoplasmic stress granules by a methylation-dependent mechanism and acts as a translational repressor. *Exp. Cell Res.*.

[7] Aoki K, Ishii Y, Matsumoto K, Tsujimoto M (2002). Methylation of Xenopus CIRP2 regulates its arginine- and glycine-rich region-mediated nucleocytoplasmic distribution. *Nucleic Acids Res.*.

[8] Wang X, Che H, Zhang W (2015). Effects of mild chronic intermittent cold exposure on rat organs. *Int. J. Biol. Sci.*.

[9] Lopez M, Meier D, Müller A, Franken P, Fujita J, Fontana A (2014). Tumor necrosis factor and transforming growth factor β regulate clock genes by controlling the expression of the cold inducible RNA-binding protein (CIRBP). *J. Biol. Chem.*.

[10] Chen X, Liu X, Li B (2017). Cold inducible RNA binding protein is involved in chronic hypoxia induced neuron apoptosis by down-regulating HIF-1α expression and regulated by microRNA-23a. *Int. J. Biol. Sci.*.

[11] Wellmann S, Bührer C, Moderegger E (2004). Oxygen-regulated expression of the RNA-binding proteins RBM3 and CIRP by a HIF-1-independent mechanism. *J. Cell. Sci.*.

[12] Liu G-P, Zhang Y, Yao X-Q (2008). Activation of glycogen synthase kinase-3 inhibits protein phosphatase-2A and the underlying mechanisms. *Neurobiol. Aging*.

[13] Pan Y, Cui Y, He H (2015). Developmental competence of mature yak vitrified-warmed oocytes is enhanced by IGF-I via modulation of CIRP during *in vitro* maturation. *Cryobiology*.

[14] Pan F, Zarate J, Choudhury A, Rupprecht R, Bradley TM (2004). Osmotic stress of salmon stimulates upregulation of a cold inducible RNA binding protein (CIRP) similar to that of mammals and amphibians. *Biochimie*.

[15] Peng Y, Kok KH, Xu RH (2000). Maternal cold inducible RNA binding protein is required for embryonic kidney formation in Xenopus laevis. *FEBS Lett.*.

[16] Matsumoto K, Aoki K, Dohmae N, Takio K, Tsujimoto M (2000). CIRP2, a major cytoplasmic RNA-binding protein in Xenopus oocytes. *Nucleic Acids Res.*.

[17] Al-Fageeh MB, Smales CM (2009). Cold-inducible RNA binding protein (CIRP) expression is modulated by alternative mRNAs. *RNA*.

[18] Sumitomo Y, Higashitsuji H, Higashitsuji H (2012). Identification of a novel enhancer that binds Sp1 and contributes to induction of cold-inducible RNA-binding protein (cirp) expression in mammalian cells. *BMC Biotechnol.*.

[19] Beishline K, Azizkhan-Clifford J (2015). Sp1 and the “hallmarks of cancer”. *FEBS J*.

[20] Jin H-O, An S, Lee H-C (2007). Hypoxic condition- and high cell density-induced expression of Redd1 is regulated by activation of hypoxia-inducible factor-1alpha and Sp1 through the phosphatidylinositol 3-kinase/Akt signaling pathway. *Cell Signal.*.

[21] Chuang J-Y, Chang W-C, Hung J-J (2011). Hydrogen peroxide induces Sp1 methylation and thereby suppresses cyclin B1 via recruitment of Suv39H1 and HDAC1 in cancer cells. *Free Radic. Biol. Med.*.

[22] Ryu H, Lee J, Zaman K (2003). Sp1 and Sp3 are oxidative stress-inducible, antideath transcription factors in cortical neurons. *J. Neurosci.*.

[23] Gotic I, Omidi S, Fleury-Olela F, Molina N, Naef F, Schibler U (2016). Temperature regulates splicing efficiency of the cold-inducible RNA-binding protein gene Cirbp. *Genes Dev*.

[24] Fujita T, Higashitsuji H, Higashitsuji H (2017). TRPV4-dependent induction of a novel mammalian cold-inducible protein SRSF5 as well as CIRP and RBM3. *Sci. Rep.*.

[25] Anderson P, Kedersha N (2009). RNA granules: post-transcriptional and epigenetic modulators of gene expression. *Nat Rev Mol. Cell Biol*.

[26] Yang R, Zhan M, Nalabothula NR, Yang Q, Indig FE, Carrier F (2010). Functional significance for a heterogenous ribonucleoprotein A18 signature RNA motif in the 3’-untranslated region of ataxia telangiectasia mutated and Rad3-related (ATR) transcript. *J. Biol. Chem.*.

[27] Yang R, Weber DJ, Carrier F (2006). Post-transcriptional regulation of thioredoxin by the stress inducible heterogenous ribonucleoprotein A18. *Nucleic Acids Res.*.

[28] Kim L, Kimmel AR (2000). GSK3, a master switch regulating cell-fate specification and tumorigenesis. *Curr. Opin. Genet. Dev.*.

[29] Pluemsampant S, Safronova OS, Nakahama K, Morita I (2008). Protein kinase CK2 is a key activator of histone deacetylase in hypoxia-associated tumors. *Int. J. Cancer*.

[30] Yang C, Carrier F (2001). The UV-inducible RNA-binding protein A18 (A18 hnRNP) plays a protective role in the genotoxic stress response. *J. Biol. Chem.*.

[31] Xia Z, Zheng X, Zheng H, Liu X, Yang Z, Wang X (2012). Cold-inducible RNA-binding protein (CIRP) regulates target mRNA stabilization in the mouse testis. *FEBS Lett.*.

[32] Liu Y, Hu W, Murakawa Y (2013). Cold-induced RNA-binding proteins regulate circadian gene expression by controlling alternative polyadenylation. *Sci. Rep.*.

[33] Coburn K, Melville Z, Aligholizadeh E (2013). Crystal structure of the human heterogeneous ribonucleoprotein A18 RNA-recognition motif. *Acta Crystallogr. Sect. F Struct. Biol. Commun.*.

[34] Haley B, Paunesku T, Protić M, Woloschak GE (2009). Response of heterogeneous ribonuclear proteins (hnRNP) to ionising radiation and their involvement in DNA damage repair. *Int. J. Radiat. Biol.*.

[35] Peng Y, Yang P-H, Tanner JA (2006). Cold-inducible RNA binding protein is required for the expression of adhesion molecules and embryonic cell movement in Xenopus laevis. *Biochem. Biophys. Res. Commun.*.

[36] Morf J, Rey G, Schneider K (2012). Cold-inducible RNA-binding protein modulates circadian gene expression posttranscriptionally. *Science*.

[37] Zhang Y, Wu Y, Mao P (2016). Cold-inducible RNA-binding protein CIRP/hnRNP A18 regulates telomerase activity in a temperature-dependent manner. *Nucleic Acids Res.*.

[38] Chang ET, Parekh PR, Yang Q, Nguyen DM, Carrier F (2012). Heterogenous ribonucleoprotein A18 (hnRNP A18) promotes tumor growth by increasing protein translation of selected transcripts in cancer cells. *Oncotarget*.

[39] Li J, Xie D, Huang J (2015). Cold-inducible RNA-binding protein regulates cardiac repolarization by targeting transient outward potassium channels. *Circ. Res.*.

[40] Aoki K, Matsumoto K, Tsujimoto M (2015). Xenopus cold-inducible RNA-binding protein 2 interacts with ElrA, the Xenopus homolog of HuR, and inhibits deadenylation of specific mRNAs. *J. Biol. Chem.*.

[41] Mukherjee N, Corcoran DL, Nusbaum JD (2011). Integrative regulatory mapping indicates that the RNA-binding protein HuR couples pre-mRNA processing and mRNA stability. *Mol. Cell*.

[42] Guo X, Wu Y, Hartley RS (2010). Cold-inducible RNA-binding protein contributes to human antigen R and cyclin E1 deregulation in breast cancer. *Mol. Carcinog.*.

[43] Tang C, Wang Y, Lan D (2015). Analysis of gene expression profiles reveals the regulatory network of cold-inducible RNA-binding protein mediating the growth of BHK-21 cells. *Cell. Biol. Int.*.

[44] Nishiyama H, Danno S, Kaneko Y (1998). Decreased expression of cold-inducible RNA-binding protein (CIRP) in male germ cells at elevated temperature. *Am. J. Pathol.*.

[45] Masuda T, Itoh K, Higashitsuji H (2012). Cold-inducible RNA-binding protein (Cirp) interacts with Dyrk1b/Mirk and promotes proliferation of immature male germ cells in mice. *Proc. Natl Acad. Sci. USA*.

[46] Nishiyama H, Xue JH, Sato T (1998). Diurnal change of the cold-inducible RNA-binding protein (Cirp) expression in mouse brain. *Biochem. Biophys. Res. Commun.*.

[47] Nikonova EV, Gilliland JD, Tanis KQ (2017). Transcriptional profiling of cholinergic neurons from basal forebrain identifies changes in expression of genes between sleep and wake: mRNA profiling in basal forebrain with sleep/sleep deprivation. *Sleep*.

[48] Oishi K, Yamamoto S, Uchida D, Doi R (2013). Ketogenic diet and fasting induce the expression of cold-inducible RNA-binding protein with time-dependent hypothermia in the mouse liver. *FEBS Open Bio*.

[49] Li X-M, Delaunay F, Dulong S (2010). Cancer inhibition through circadian reprogramming of tumor transcriptome with meal timing. *Cancer Res.*.

[50] Hamid AA, Mandai M, Fujita J (2003). Expression of cold-inducible RNA-binding protein in the normal endometrium, endometrial hyperplasia, and endometrial carcinoma. *Int. J. Gynecol. Pathol.*.

[51] Lujan DA, Garcia S, Vanderhoof J (2016). Cold-inducible RNA binding protein in mouse mammary gland development. *Tissue Cell*.

[52] Zhang Q, Wang Y-Z, Zhang W (2017). Involvement of cold inducible RNA-binding protein in severe hypoxia-induced growth arrest of neural stem cells *in vitro*. *Mol. Neurobiol.*.

[53] Hong JK, Kim Y-G, Yoon SK, Lee GM (2007). Down-regulation of cold-inducible RNA-binding protein does not improve hypothermic growth of Chinese hamster ovary cells producing erythropoietin. *Metab. Eng.*.

[54] Tan HK, Lee MM, Yap MGS, Wang DIC (2008). Overexpression of cold-inducible RNA-binding protein increases interferon-gamma production in Chinese-hamster ovary cells. *Biotechnol. Appl. Biochem.*.

[55] Van Venrooy S, Fichtner D, Kunz M, Wedlich D, Gradl D (2008). Cold-inducible RNA binding protein (CIRP), a novel XTcf-3 specific target gene regulates neural development in Xenopus. *BMC Dev. Biol.*.

[56] Uochi T, Asashima M (1998). XCIRP (Xenopus homolog of cold-inducible RNA-binding protein) is expressed transiently in developing pronephros and neural tissue. *Gene*.

[57] Lee HN, Ahn S-M, Jang HH (2015). Cold-inducible RNA-binding protein, CIRP, inhibits DNA damage-induced apoptosis by regulating p53. *Biochem. Biophys. Res. Commun.*.

[58] Banks S, King SA, Irvine DS, Saunders PTK (2005). Impact of a mild scrotal heat stress on DNA integrity in murine spermatozoa. *Reproduction*.

[59] Xia Z-P, Zheng X-M, Zheng H, Liu X-J, Liu G-Y, Wang X-H (2012). Downregulation of cold-inducible RNA-binding protein activates mitogen-activated protein kinases and impairs spermatogenic function in mouse testes. *Asian J. Androl.*.

[60] Long TY, Jing R, Kuang F, Huang L, Qian ZX, Yang TL (2017). CIRBP protects H9C2 cells against myocardial ischemia through inhibition of NF-κB pathway. *Braz. J. Med. Biol. Res.*.

[61] Zhou K-W, Zheng X-M, Yang Z-W, Zhang L, Chen H-D (2009). Overexpression of CIRP may reduce testicular damage induced by cryptorchidism. *Clin. Invest. Med.*.

[62] Xia Z, Jiang K, Liu T, Zheng H, Liu X, Zheng X (2013). The protective effect of cold-inducible RNA-binding protein (CIRP) on testicular torsion/detorsion: an experimental study in mice. *J. Pediatr. Surg.*.

[63] Artero-Castro A, Callejas FB, Castellvi J (2009). Cold-inducible RNA-binding protein bypasses replicative senescence in primary cells through extracellular signal-regulated kinase 1 and 2 activation. *Mol. Cell Biol.*.

[64] Sakurai T, Itoh K, Higashitsuji H (2006). Cirp protects against tumor necrosis factor-alpha-induced apoptosis via activation of extracellular signal-regulated kinase. *Biochim. Biophys. Acta*.

[65] Saito K, Fukuda N, Matsumoto T (2010). Moderate low temperature preserves the stemness of neural stem cells and suppresses apoptosis of the cells via activation of the cold-inducible RNA binding protein. *Brain Res.*.

[66] Li S, Zhang Z, Xue J, Liu A, Zhang H (2012). Cold-inducible RNA binding protein inhibits H_2_O_2_-induced apoptosis in rat cortical neurons. *Brain Res.*.

[67] Liu J, Xue J, Zhang H (2015). Cloning, expression, and purification of cold inducible RNA-binding protein and its neuroprotective mechanism of action. *Brain Res.*.

[68] Wu L, Sun H-L, Gao Y (2016). Therapeutic hypothermia enhances cold-inducible RNA-Binding protein expression and inhibits mitochondrial apoptosis in a rat model of cardiac arrest. *Mol. Neurobiol.*.

[69] Wang G, Zhang J-N, Guo J-K (2016). Neuroprotective effects of cold-inducible RNA-binding protein during mild hypothermia on traumatic brain injury. *Neural Regen. Res.*.

[70] Meeker AK, Coffey DS (1997). Telomerase: a promising marker of biological immortality of germ, stem, and cancer cells. A review. *Biochemistry Mosc.*.

[71] Park BM, Lee JH, Kim SJ (2014). Expression of cold-inducible RNA-binding protein in normal skin, actinic keratosis and squamous cell carcinoma. *Ann. Dermatol.*.

[72] Harris AL (2002). Hypoxia--a key regulatory factor in tumour growth. *Nat. Rev. Cancer*.

[73] Ren WH, Zhang LM, Liu HQ (2014). Protein overexpression of CIRP and TLR4 in oral squamous cell carcinoma: an immunohistochemical and clinical correlation analysis. *Med. Oncol.*.

[74] Wang M, Zhang H, Heng X, Pang Q, Sun A (2015). Expression of cold-inducible RNA-binding protein (CIRP) in pituitary adenoma and its relationships with tumor recurrence. *Med. Sci. Monit.*.

[75] Mangé A, Lacombe J, Bascoul-Mollevi C (2012). Serum autoantibody signature of ductal carcinoma *in situ* progression to invasive breast cancer. *Clin. Cancer Res.*.

[76] Liao Y, Feng J, Zhang Y, Tang L, Wu S (2017). The mechanism of CIRP in inhibition of keratinocytes growth arrest and apoptosis following low dose UVB radiation. *Mol. Carcinog.*.

[77] Jian F, Chen Y, Ning G (2016). Cold inducible RNA binding protein upregulation in pituitary corticotroph adenoma induces corticotroph cell proliferation via Erk signaling pathway. *Oncotarget*.

[78] Lee HN, Ahn S-M, Jang HH (2016). Cold-inducible RNA-binding protein promotes epithelial-mesenchymal transition by activating ERK and p38 pathways. *Biochem. Biophys. Res. Commun.*.

[79] He H, Altomare D, Ozer U (2016). Cancer cell-selective killing polymer/copper combination. *Biomater. Sci.*.

[80] Zeng Y, Kulkarni P, Inoue T, Getzenberg RH (2009). Down-regulating cold shock protein genes impairs cancer cell survival and enhances chemosensitivity. *J. Cell Biochem.*.

[81] Lleonart ME (2010). A new generation of proto-oncogenes: cold-inducible RNA binding proteins. *Biochim. Biophys. Acta*.

[82] Brochu C, Cabrita MA, Melanson BD (2013). NF-κB-dependent role for cold-inducible RNA binding protein in regulating interleukin 1β. *PLoS ONE*.

[83] Qiang X, Yang W-L, Wu R (2013). Cold-inducible RNA-binding protein (CIRP) triggers inflammatory responses in hemorrhagic shock and sepsis. *Nat Med*.

[84] Li Z, Fan EK, Liu J (2013). Cold-inducible RNA-binding protein through TLR4 signaling induces mitochondrial DNA fragmentation and regulates macrophage cell death after trauma. *Cell Death Dis.*.

[85] Yang W-L, Sharma A, Wang Z, Li Z, Fan J, Wang P (2016). Cold-inducible RNA-binding protein causes endothelial dysfunction via activation of Nlrp3 inflammasome. *Sci. Rep.*.

[86] Khan MM, Yang W-L, Brenner M, Bolognese AC, Wang P (2017). Cold-inducible RNA-binding protein (CIRP) causes sepsis-associated acute lung injury via induction of endoplasmic reticulum stress. *Sci. Rep.*.

[87] Bolognese AC, Sharma A, Yang W-L, Nicastro J, Coppa GF, Wang P (2016). Cold-inducible RNA-binding protein activates splenic T cells during sepsis in a TLR4-dependent manner. *Cell Mol. Immunol.*.

[88] Zhou Y, Dong H, Zhong Y, Huang J, Lv J, Li J (2015). The cold-inducible RNA-binding protein (CIRP) level in peripheral blood predicts sepsis outcome. *PLoS ONE*.

[89] Zhang F, Yang W-L, Brenner M, Wang P (2017). Attenuation of hemorrhage-associated lung injury by adjuvant treatment with C23, an oligopeptide derived from cold-inducible RNA-binding protein (CIRP). *J. Trauma Acute Care Surg.*.

[90] Rajayer SR, Jacob A, Yang W-L, Zhou M, Chaung W, Wang P (2013). Cold-inducible RNA-binding protein is an important mediator of alcohol-induced brain inflammation. *PLoS ONE*.

[91] Zhou M, Yang W-L, Ji Y, Qiang X, Wang P (2014). Cold-inducible RNA-binding protein mediates neuroinflammation in cerebral ischemia. *Biochim. Biophys. Acta*.

[92] Godwin A, Yang W-L, Sharma A (2015). Blocking cold-inducible RNA-binding protein protects liver from ischemia-reperfusion injury. *Shock*.

[93] Cen C, Yang W-L, Yen H-T, Nicastro JM, Coppa GF, Wang P (2016). Deficiency of cold-inducible ribonucleic acid-binding protein reduces renal injury after ischemia-reperfusion. *Surgery*.

[94] Li G, Yang L, Yuan H (2016). Cold-inducible RNA-binding protein plays a central role in the pathogenesis of abdominal aortic aneurysm in a murine experimental model. *Surgery*.

[95] Ran D, Chen L, Xie W (2016). Cold-inducible RNA binding protein regulates mucin expression induced by cold temperatures in human airway epithelial cells. *Arch. Biochem. Biophys.*.

[96] Chen L, Ran D, Xie W, Xu Q, Zhou X (2016). Cold-inducible RNA-binding protein mediates cold air inducible airway mucin production through TLR4/NF-κB signaling pathway. *Int. Immunopharmacol.*.

[97] Yu L, Li Q-H, Deng F, Yu Z-W, Luo X-Z, Sun J-L (2017). Synovial fluid concentrations of cold-inducible RNA-binding protein are associated with severity in knee osteoarthritis. *Clin. Chim. Acta*.

[98] Idrovo JP, Jacob A, Yang WL (2016). A deficiency in cold-inducible RNA-binding protein accelerates the inflammation phase and improves wound healing. *Int. J. Mol. Med.*.

[99] Sakurai T, Yada N, Watanabe T (2015). Cold-inducible RNA-binding protein promotes the development of liver cancer. *Cancer Sci.*.

[100] Sakurai T, Kashida H, Watanabe T (2014). Stress response protein cirp links inflammation and tumorigenesis in colitis-associated cancer. *Cancer Res.*.

