# Corrigendum

**DOI:** 10.4155/fsoa-2017-0077c1

**Published:** 2018-04-11

In the Review by, Peng Zhong & HE Huang, ‘Recent progress in the research of cold-inducible RNA-binding protein’, which appeared in October 2017 in *Future Science OA*, 3(4), FSO246 (2017), there was a misspelling in affiliation 1. This was published incorrectly as: Department of Cardiology, Renming Hospital of Wuhan University, Wuhan 430060, PR China. The correct affiliation is: Department of Cardiology, Renmin Hospital of Wuhan University, Wuhan 430060, PR China.

The authors and editors of *Future Science OA* would like to sincerely apologize for any inconvenience or confusion this may have caused our readers.

